# Replication-Fork Stalling and Processing at a Single Psoralen Interstrand Crosslink in Xenopus Egg Extracts

**DOI:** 10.1371/journal.pone.0018554

**Published:** 2011-04-15

**Authors:** Cyrille Le Breton, Magali Hennion, Paola B. Arimondo, Olivier Hyrien

**Affiliations:** 1 Institut de Biologie de l'Ecole Normale Supérieure, CNRS UMR 8197-Inserm U1024, Paris, France; 2 Museum National d'Histoire Naturelle UMR 7196 CNRS-INSERM U565, Paris, France; University of Minnesota, United States of America

## Abstract

Interstrand crosslink (ICL)-inducing agents block the separation of the two DNA strands. They prevent transcription and replication and are used in clinics for the treatment of cancer and skin diseases. Here, we have introduced a single psoralen ICL at a specific site in plasmid DNA using a triplex-forming-oligonucleotide (TFO)-psoralen conjugate and studied its repair in Xenopus egg extracts that support nuclear assembly and replication of plasmid DNA. Replication forks arriving from either side stalled at the psoralen ICL. In contrast to previous observations with other ICL-inducing agents, the leading strands advanced up to the lesion without any prior pausing. Subsequently, incisions were introduced on one parental strand on both sides of the ICL. These incisions could be detected whether one or both forks reached the ICL. Using small molecule inhibitors, we found that the ATR-Chk1 pathway, but not the ATM-Chk2 pathway, stimulated both the incision step and the subsequent processing of the broken replication intermediates. Our results highlight both similarities and differences in fork stalling and repair induced by psoralen and by other ICL-forming agents.

## Introduction

Covalent DNA interstrand crosslinks (ICLs) block the separation of the two DNA strands required for transcription and replication of the genetic material. ICL-inducing agents such as psoralen with ultraviolet (UV) light, mitomycin C, nitrogen mustards and cisplatin are therefore particularly toxic, especially in proliferating cells, and are largely used in the treatment of cancers and skin diseases [1]. ICL-inducing agents are also produced during cellular lipid peroxidation [2]. Both exogenous and endogenous sources of ICL seem to contribute to aging [3].

ICLs pose a challenge to repair because both DNA strands are damaged. Studies of DNA-repair defective cell lines have shown that various proteins implicated in nucleotide excision repair (NER), homologous recombination (HR), translesion DNA synthesis and Fanconi anemia (FANC) participate in the detection and repair of ICLs [4], [5]. The proposed steps of ICL repair involve i) the generation of incisions on both sides of the lesion by structure-specific endonucleases such as ERCC1/XPF [6], MUS81/EME1 [7] and the newly described FAN1 5′ exonuclease/flap endonuclease [8], [9], [10], followed by unhooking of the adduct; ii) the extension of the 3′ end generated during the incision through the remaining monoadduct by translesion DNA polymerases such as REV1 and polymerase ζ [11], [12], or polymerase η [13], or polymerase κ [14]; and iii) the removal of the remaining monoadduct by NER proteins [15] or by the DNA glycosylase NEIL1 [16]. When repair occurs at a stalled replication fork by the ICL, the incisions result in a double strand break (DSB) and release of one of the replicated sister chromatids, which is then restored by HR using the unbroken sister chromatid as homology donor. FANC proteins have been proposed to regulate the incision and translesion steps as well as HR and to participate in checkpoint signaling in response to ICLs [5].

Xenopus egg extracts have been used to study the repair of a single ICL in plasmid DNA [17]. Raschle et al. [18] defined molecular details of replication-dependent repair of nitrogen-mustard like and cisplatin-induced crosslinks. They showed that two replication forks converge on the ICL with their leading strands initially stalling 20 nt (cisplatin) or 24 nt (nitrogen mustard-like) from the lesion. Subsequently, one of the two leading strands advances to within 1 nt from the ICL before FANCD2/I-dependent incisions on the other parental strand uncouple the two sister chromatids. Lesion bypass then occurs by FANCD2/I-dependent nucleotide insertion across the damaged template base followed by polymerase ζ-dependent extension. Raschle et al. also reported that Chk1 is phosphorylated and FANCD2 is ubiquitylated in a strictly replication-dependent manner during this process. In contrast, using the same experimental system Ben-Yehoyada et al. [19] reported that mitomycin C-induced ICLs trigger a checkpoint response independently of origin initiated DNA replication. These authors suggested that the Fanconi anemia pathway acts upstream of RPA-ATR-Chk1 to generate the ICL signal.

Studies in various experimental systems indicate that details of the cellular response to ICLs can depend on the ICL type. For example, in yeast, nucleotide excision repair pathway has been implicated in the generation of DSBs in response to psoralen ICLs [20], [21] but not to nitrogen mustard-DNA adducts [22]. Here, we have used a triplex-forming-oligonucleotide (TFO)-psoralen conjugate to introduce a psoralen ICL at a specific site in plasmid DNA. We have studied the replication-coupled repair of this site-specific ICL in Xenopus egg extracts that support chromatinization and nuclear-assembly dependent replication of plasmid DNA. The results show that both fork stalling and incision differ from other ICLs and that the ATR-Chk1 pathway stimulates both incision and following steps leading to the final repair product.

## Results

### Purification of a plasmid containing a site-specific psoralen interstrand crosslink

**Figure 1:**
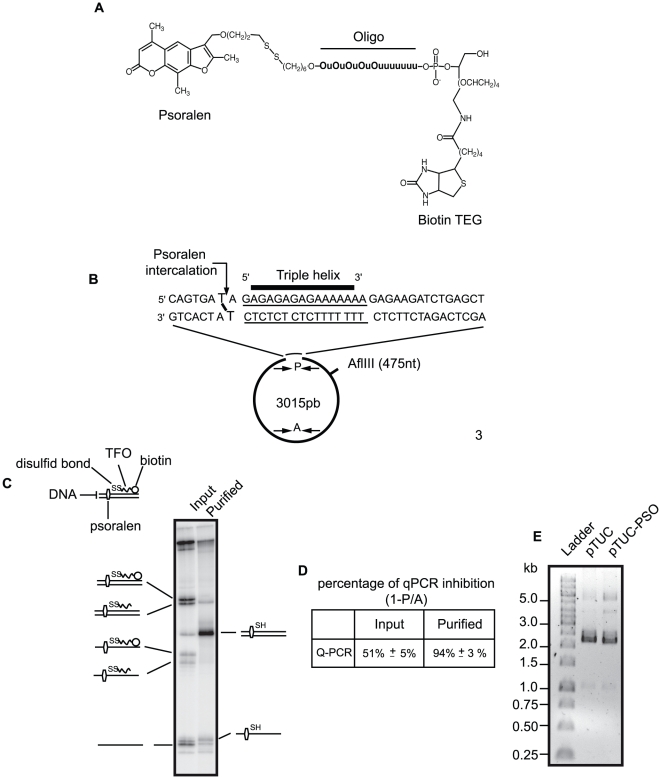
TFO and purification of monomodified plasmid. (A) Structure of the TFO linked in 3′ to 4, 5′, 8-trimethylpsoralen and in 5′ to biotin. (B) Localization of the TFO binding site and position of the psoralen ICL on pTUC plasmid. P and A are schematic representations of primers for q-PCR used in (D). P primers surround the psoralen ICL site and amplify a region of 113 nt, the A primers amplify an undamaged regions of 129 nt. (C) Analysis of the crosslinked plasmid after UV irradiation and before (input) or after (purified) DTT elution from a strepatvidin column. DNA was digested with BamHI + EcoRI and radioactively labelled before electrophoresis on a 10% polyacrylamide denaturing gel. Interpretative diagrams of the relevant molecular species are shown on lane sides. (D) Input and purified plasmids were subjected to q-PCR with primers P and A. Mean values of 3 q-PCRs for the input and 12 q-PCRs for the purified plasmid are presented. For calculation details see Material and Methods. (E) After purification pTUC-PSO was analyzed on a 0.8% agarose gel TBE 1x alongside with control plasmid and a molecular weight ladder.

Triplex-forming oligonucleotides (TFO) conjugates are widely used to introduce DNA lesions at specific sites in plasmids or in genomic DNA [23],[24]. Since triplex DNA alone has been reported to interfere with DNA repair [25], [26], we devised a method to eliminate the TFO moiety after introducing a psoralen crosslink at a specific site in the pTUC plasmid. The TFO conjugate used in our study is described in Figure 1A. The TFO moiety contains 5-methyldeoxycytosine (O) and 5-propynyldeoxyuridine (u) bases to increase triplex formation [27]. The TFO moiety is linked in 5′ through a scissile S-S bond to 4,5′,8-trimethylpsoralen and in 3′ to biotin TEG. The TFO binds a unique sequence on the pTUC plasmid that allows us to position the psoralen moiety at a defined site between two AT base pairs as shown on Figure 1B. The psoralen DNA interstrand crosslink (ICL) was generated by 310 nm UV irradiation, which favours production of ICLs over monoadducts. The crosslinked plasmid was bound to a streptavidin column and eluted with dithiotreitol (DTT) that reduces the disulfide bond between the psoralen and the TFO. The structure of the targeted site before and after column purification was assessed by restriction enzyme digestion, fill-in radioactive labeling and polyacrylamide-urea gel electrophoresis (Fig. 1C). Gel scanning indicated that 50% of the input and 81% of the eluted plasmid contained a psoralen ICL, the rest being a mixture of intact and monoadducted molecules. The gel also showed that most of the eluted crosslinked plasmid had lost the TFO moiety, as expected. We also quantified the extent of plasmid modification by q-PCR, as the DNA polymerase cannot synthesize DNA across a psoralen lesion (Fig. 1D). The amplification signal obtained with a primer pair flanking the psoralen adduct was 49% (input plasmid) and 6% (eluted plasmid) of that obtained with primers flanking a control region, consistent with the gel quantitation results. The purified crosslinked plasmid migrated on an agarose gel in an indistinguishable manner from the control supercoiled plasmid (Fig. 1E).

### Replication of the psoralen-crosslinked plasmid in Xenopus egg extract

We studied the effect of the site-specific psoralen ICL on replication fork progression and repair in low speed supernatants (LSS) from Xenopus eggs. Plasmid DNA incubated in these extracts undergoes chromatinisation and assembly into pseudonuclei followed by one cycle of semiconservative DNA replication [28], [29]. Plasmid DNA supports a single, randomly located initiation event in this system [30]. The ICL-containing plasmid (pTUC-PSO) or the control plasmid (pTUC) was incubated in the presence of [α-^32^P]-dATP, purified, digested with AflIII and analysed by two-dimensional (2D) agarose gel electrophoresis, a technique that allows to resolve replication-fork containing restriction fragments according to their mass and shape.

**Figure 2:**
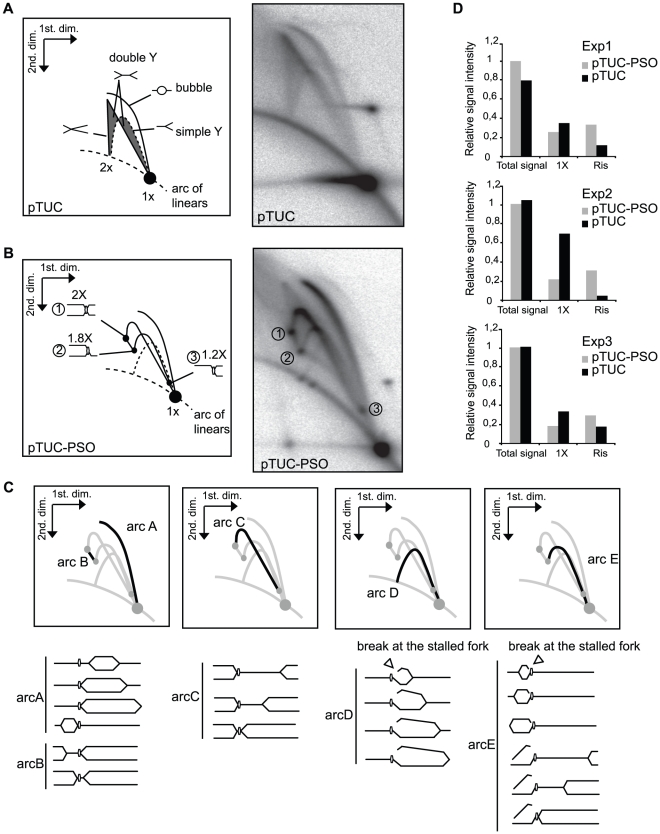
Two-dimensional gel electrophoretic analysis of pTUC and pTUC-PSO plasmids replicating in Xenopus egg extracts. Plasmids pTUC (A) and pTUC-PSO (B) were incubated 95 min in Xenopus egg extract in the continuous presence of [α-^32^P]-dATP. Plasmids were purified, linearized by AflIII digestion and analyzed by 2D gel electrophoresis. Phosphorimager images of the dried gels and interpretative diagrams are shown. (C) Interpretation of the pTUC-PSO specific arcs. (D) Relative intensity of total signal, 1x spot and replication intermediates (RIs) from 3 independent experiments (Exp1, Exp2, Exp3).

Since pTUC DNA contains a single AflIII site and replication initiates at random, replication intermediates (RIs) were expected to contain two replication forks in either divergent (bubble) or convergent (double-Y) orientation, with all possible geometries. Indeed, a complete bubble arc and a complete pattern of symmetric and asymmetric double Ys were observed (Fig. 2A). A radio-labeled linear fragment corresponding to the fully replicated plasmid (1× spot) was also detected.

The 2D-gel pattern of pTUC-PSO was different (Fig. 2B). AflIII cuts the plasmid so that the psoralen ICL divides the fragment in two parts comprising 80% and 20% of its length, respectively. The bubble arc (arcA, Fig. 2C) did not reach the 2x position but stopped abruptly at the 1.8x position, suggesting that a fork initiated in the large part of the AflIII fragment was stalled at the ICL. The missing portion of the bubble arc was replaced by a short arc of asymmetric double Ys (arc B, Fig. 2C) ranging from 1.8x to 2x relative mass and spreading between two spots labeled 1 and 2 on Figure 2B. We interpret this as a bubble-to double-Y transition caused by the stalling of the fork arriving at the ICL. We also expected a similar bubble-to double-Y transition happening on the other side of the ICL. This should generate bubbles ranging from the 1.0x to 1.2 x mass (contributing to the lower portion of the bubble arc) and asymmetric double Ys ranging from 1.2x to 2.0x mass (arc C, Fig. 2C), as observed (Fig. 2B). This arc shows a prominent hook and is clearly different from the smear of double Ys generated by pTUC. This is because one fork is small and stays stationary at the ICL while the other one moves, so that the shape and electrophoretic behavior of the double Y increasingly mimic a simple Y, as previously described [31]. A prominent spot of accumulated asymmetric double Ys of mass 2.0x was observed (spot 1), suggesting that the two forks, which converged at the ICL, remained stalled there for some time. An accumulation of Y-shaped molecules at the 1.2x (spot 3) and 1.8x (spot 2) positions was also observed (Fig. 2B). Since AflIII cuts the plasmid only once, RIs with a single fork were not expected unless fork breakage occurred on a two-forked RI. We therefore interpret spot 2 and spot 3 as due to breakage of either of the two stalled forks at spot 1 molecules. We wondered whether fork breakage required that both forks were stalled at the ICL or could also occur when one fork was stalled and the other fork was still moving. In this case, breakage of the stalled fork located on the large part of the AflIII fragment should result in a series of asymmetric Y molecules (arcD, Fig. 2C) similar though not identical to the population of molecules that generate a simple Y pattern, as demonstrated elsewhere [32]. The signal produced would return to the arc of linear forms at a mass equivalent of 1.8x rather than 2.0x. A faint arc with these characteristics was indeed observed (Fig. 2B). Breakage of the stalled fork on the small part of the AflIII fragment should result in a series of symmetric Y molecules (arcE, Fig. 2C) that should generate a typical simple Y pattern, but the signal produced would end at the 1.8x position before reaching the diagonal of linear fragments. This arc was also observed (Fig. 2B). Note that breakage of the two kinds of stalled forks is also expected to produce linear fragments of relative mass 0.8x and 0.2x, respectively, which were observed (these fragments ran out of the gel portion shown on Figure 2 but the 0.8x fragment is visible on further 2D-gel of checkpoint experiments). We conclude that the psoralen ICL stalls forks arriving from both sides and that fork breakage can occur on either side of the ICL whether one or both forks have reached the ICL. Importantly, a linear fragment corresponding to the fully replicated plasmid (1× spot) was also detected. Complete replication of contaminating unmodified plasmid may have contributed to this 1x spot. However, this contribution must be minor because specific RIs of the unmodified plasmid migrating at the top (>1.8 x) portion of the bubble arc and between spot 2 and the arc of linear molecules (Fig. 2A) were not detected on Figure 2B (see also further 2D-gel of checkpoint experiments). This result also suggests that no significant repair of the ICL occured prior to replication. Therefore, most of the 1x spot signal must come from the modified plasmid. This implies a complete segregation of the two parental template strands, *i.e.*, a double incision of one parental strand around the ICL (unhooking). We found no evidence for dual incision of both parental strands. This pattern of incisions would have resulted in a 2x linear molecule, which was not observed. We conclude that although the first arrived fork can be broken before the arrival of the second fork, incisions occur in a concerted manner so that only one parental strand is broken at a time. As will be shown below, the continuity of the daughter strand is reestablished following this step.

There is a controversy in the DNA repair field as to whether replication forks stall and retreat once they meet a DNA lesion. A triangular smear was observed above the upper part of arc C generated by pTUC-PSO. This signal may correspond to branch-migrated intermediates from arcs B and C [33], [34]. Alternatively, they may correspond to random termination intermediates. A careful comparison of the triangular smear of pTUC-PSO with the random termination intermediates generated by pTUC shows a lack of double Ys below arc B, inconsistent with random termination even in a small fraction of pTUC-PSO molecules. The abrupt ending of the bubble arc at the 1.8x position is also inconsistent with this hypothesis. Therefore, we do not exclude that a small fraction of stalled forks at the ICL undergo regression to contribute to this triangular smear.

To look whether the global level of plasmid replication was affected by the ICL, we quantified the total radioactivity incorporated in plasmid DNA. A similar level of replication was observed for the control and the ICL plasmid (Fig. 2D, total signal). However, the proportion of RIs and 1x spot were different. The amount of RIs was increased on average by 2.6-fold and the 1x spot was decreased on average by 2.1-fold in the presence of the ICL as compared with the control (average of 3 independent experiments presented in Figure 2D). Thus, both the turnover of RIs and the completion of plasmid synthesis were slower in the presence of the ICL. This slower replication can be simply explained by the fact that one or both forks were stalled on most of the plasmid RIs. These data also suggest that if the stalled forks activate a checkpoint, this does not strongly downregulate replication initiation of other plasmid molecules in these experimental conditions.

Similar results (data not shown) were observed when the psoralen crosslink was introduced using a TFO-psoralen conjugate that does not allow elimination of the TFO moiety. In contrast, normal replication intermediates were observed when the plasmid was preincubated with the TFO alone (data not shown). These results confirm that fork stalling and breakage is specifically due to the psoralen ICL and show that triple helix formation alone does not prevent replication fork progression in Xenopus egg extracts.

### Leading strands advance to the ICL without pausing

**Figure 3:**
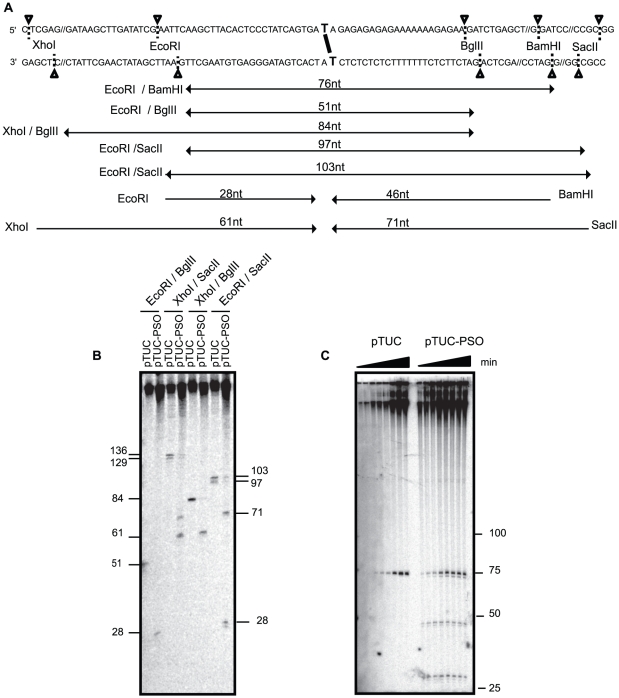
Replication fork leading strands progress up to the psoralen crosslink. (A) Sequence of the plasmid around the psoralen ICL with restriction sites used in B and C. Double arrows indicate the size of the replicated strands (lagging or leading) spanning the ICL site for the control or repaired plasmid. Single arrows indicate the progression of the leading strand to the ICL for the crosslinked plasmid. The strand size is shown above each product. (B) Mapping of leading strand progression for pTUC and pTUC-PSO after 65 min of incubation in Xenopus egg extracts in the continuous presence of [α-^32^P]-dATP. Plasmids were digested by the indicated enzymes and analyzed on 10% polyacrylamide denaturing gel. Faint bands visible in lanes 3 and 4 likely result from a star activity of Sac II. The two 28 nt size indicators are at two different positions due to gel smiling. (C) Mapping of leading strand progression for pTUC and pTUC-PSO at 25, 35, 50, 65, 85, 95, 120 and 180 min. Plasmids were digested with EcoRI and BamHI and subjected to migration on a 10% polyacrylamide denaturing gel. 28 nt and 46 nt fragments correspond to the DNA size expected for the leading strand arriving at the psoralen from the EcoRI side or BamHI side, respectively.

The 2D gels suggest that replication forks progress up to the psoralen ICL and stall there. To map more precisely the progression of the replication forks we digested the radio-labeled replicated plasmids pTUC and pTUC-PSO by different restriction enzymes as shown in Figure 3A and resolved the labeled single-strands by denaturing polyacrylamide-urea gel electrophoresis. With the control plasmid, the expected full-size labeled fragments were observed in all restriction digestion conditions (Fig. 3B). The fragment sizes observed with the ICL plasmid were all consistent with the leading strand coming from either direction being stalled at or within 1 nt of the psoralen ICL but not elsewhere. The EcoRI/BglII digestion resulted in detection of a 28 nt fragment, a size that could only correspond to the leading strand extending from the EcoRI site to the ICL. Note that because of the lack of thymine residues in the template strand, a leading strand extending from the BglII site to the ICL would not be labeled with radioactive dATP and would not be detectable. The lack of any other accumulated small fragment in this lane shows that lagging strands either did not reach the EcoRI-BglII fragment or paused at too many different sites to accumulate to a detectable level. Therefore, the 61 nt and 71 nt fragments detected upon XhoI/SacII digestion can only correspond to both leading strands arrested at the ICL, and the lack of any other fragment confirms the lack of detectable pausing site for lagging strand synthesis on both sides of the ICL. The specific accumulation of the 61 nt band in the XhoI/BglII lane and the 71 nt band in the EcoRI/SacII lane confirm these interpretations. The radio-labeled replicated plasmids were also digested with PvuII, which cuts 203 nt and 302 nt away from the ICL on the left and right side, respectively (data not shown). Although leading strands paused at the ICL were again detected, no other bands that may correspond to lagging strands arrested at a specific site were observed. Elongating lagging strands most likely pause at many positions over the examined region. The lack of detectable leading strand pausing prior to reaching the ICL is in contrast to previous results for cisplatin and nitrogen mustard-like ICLs, where leading strands were observed to initially stall at 20–24 nt from the ICL before advancing to it [18]. Overall, these results suggest that the psoralen ICL caused the replisome to pause only when the leading strand polymerase reached the lesion.

We performed a kinetic analysis to follow the fate of the arrested leading strands (Fig. 3C). pTUC and pTUC-PSO were incubated in egg extracts in the presence of [α-^32^P]-dATP and aliquots were taken at different times then digested with EcoRI + BamHI and the labeled single-strands resolved by denaturing polyacrylamide-urea gel electrophoresis. The control plasmid showed a progressive accumulation of labeled single-strand at the expected size (74 nt). The ICL plasmid additionally showed 28 nt and 46 nt accumulated strands, which correspond to the arrested leading strands from stalled forks in both orientations. Note that a fraction of leading strands was also found to accumulate 1–2 nt before reaching the ICL, as shown by the faint bands below the 46 nt and 28 nt positions. Arrested leading strands reached a steady-state level whereas the fully replicated 74 nt strand accumulated progressively, suggesting that the arrested leading strands were rapidly converted to full-size daughter strands, presumably due to lesion bypass [17]. We also noted the parallel accumulation of a weak 73 nt band, which may represent a 1 nt-deletion product formed during lesion bypass.

**Figure 4:**
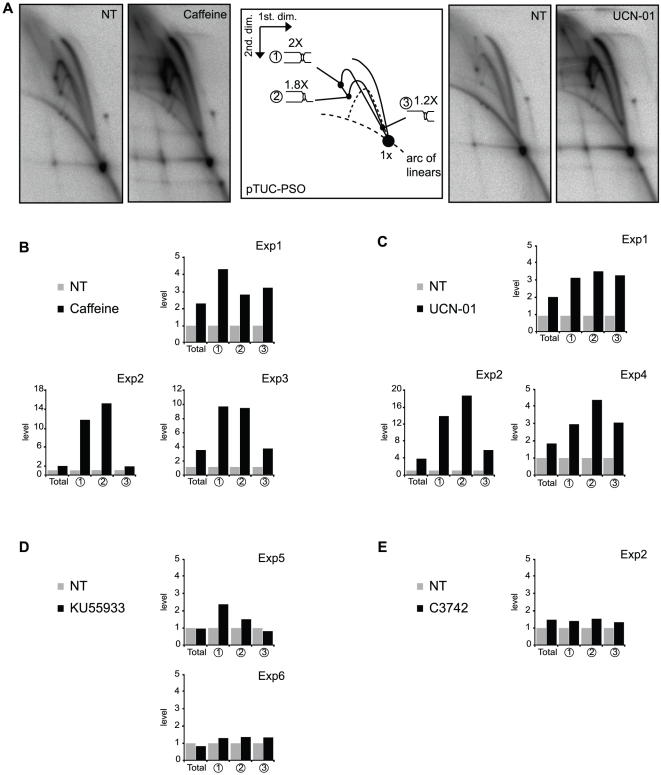
Effect of ATR and Chk1 inhibition on processing of stalled replication forks at the psoralen ICL. (A) pTUC-PSO was incubated for 95 min in a Xenopus egg extract in absence (NT; not treated) or presence of 5 mM caffeine or 10 µM UCN-01 and [α-^32^P]-dATP and analyzed on 2D gels. Total signal and spots 1, 2 and 3 (as labeled on the interpretative diagram) were quantified in the presence of caffeine (B), UCN-01 (C), KU55933 (D) or C3742 (E). Results were normalized to the signals obtained with the non-treated plasmid. Exp1, Exp2, Exp3, Exp4, Exp5 and Exp6 are six experiments in six independently prepared egg extracts.

*The psoralen ICL activates the ATR-Chk1 checkpoint.* We used inhibitors of the ATM, ATR, Chk1 and Chk2 kinases to study the effect of replication and DNA damage checkpoints on the replication and repair of the psoralen ICL-containing plasmid. The ICL-containing plasmid was incubated in the presence of [α-^32^P]-dATP plus or minus caffeine, a widely used ATM/ATR inhibitor, digested with AflIII and analysed by 2D-gel electrophoresis (Fig. 4A). The total radioactivity incorporated in plasmid DNA was increased 2-fold by caffeine (average of 4 independent experiments), but this was also observed in the absence of ICLs (Marheineke 2004, and data not shown). However, changes were also observed in the relative abundance of the different RIs in the presence of caffeine. Whereas spots 1, 2 and 3 increased 2 to 6-fold, the 1x spot did not increase (4 independent experiments shown on Fig. 4B). The accumulation of both stalled intermediates and their breakage products suggests that both fork breakage and repair were slower in the presence of caffeine, consistent with checkpoint involvement in ICL processing [35], [36]. Quantitatively similar results were observed in the presence of UCN-01, a specific Chk1 inhibitor (Fig. 4 A,C). In contrast, no significant change was observed in the presence the specific ATM inhibitor KU55933 or the specific Chk2 inhibitor C3742 (Fig. 4D,E). We conclude that the psoralen ICL activates the ATR-Chk1 pathway to stimulate breakage and repair of the stalled replication forks at the lesion.

## Discussion

**Figure 5:**
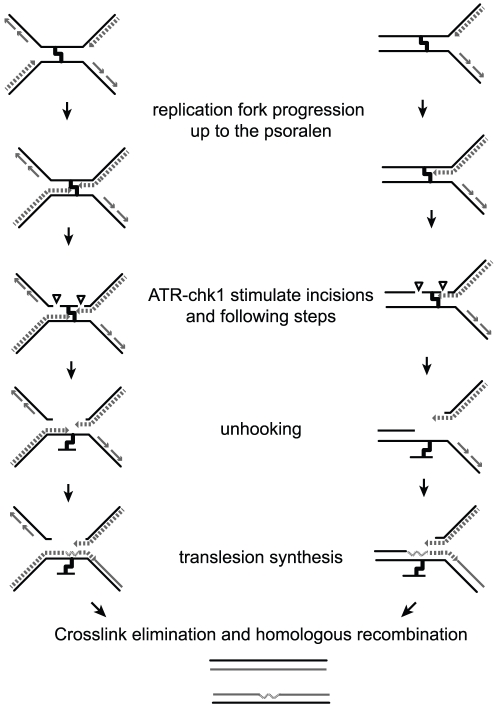
Model for psoralen ICL repair in Xenopus egg extract. During replication of plasmid DNA containing a single psoralen ICL, replication forks stall at the ICL advancing up to 1 nt from the lesion. Coordinated incisions of one parental strand around the lesion can occur whether one (right) or both (left) forks have stalled, resulting in unhooking of the lesion. Next, translesion synthesis occurs across the unhooked psoralen adduct and other events implying HR and/or NER regenerate two repaired duplexes as proposed elsewhere [5]. Both incision events and subsequent processing of the broken replication intermediates are stimulated by the ATR-Chk1 pathway.

Triplex-forming oligonucleotides have been used to specifically introduce DNA lesions at a selected target sequences. Here we devised a method to eliminate the TFO moiety after introducing a psoralen interstrand crosslink at a specific site in a plasmid DNA molecule and to study the fate of this lesion during DNA replication in Xenopus egg extracts. Our results are summarized on Figure 5.

We observed that replication forks approaching the psoralen ICL from either direction stalled at the lesion with their leading strand arrested 1–2 nt from the crosslinked base, then broke. Fork breakage occurred on either side of the ICL. Breakage of the first arrived fork before the second fork converged to the ICL was detected. In this case, incisions were fast, given that the time required for the second fork to circle around the plasmid was brief (< ∼7 min; [30]). Breakage was also observed after the two forks converged at the ICL. In this case incisions were slower since a significant amount of stalled X-shaped structures accumulated. Whether incisions occurred after one or both forks reached the ICL, they were located in only one parental strand, resulting in an unhooking intermediate. Several endonucleases have been shown (Mus81; XPF/ERCC1; FAN1) [37] [8], [9], [10] or proposed (Slx1) [38] to perform these incisions. It is possible that different nucleases act on single and double fork-stalled intermediates. Following the unhooking step, the leading strand was extended across the ICL, generating a full-length monomer plasmid. Inhibition of the ATR-Chk1 pathway slowed down both fork breakage and repair, suggesting that this pathway stimulates several steps of the lesion processing during DNA replication.

Xenopus egg extracts have recently been used to study the repair of other types of ICLs during DNA replication [18], [19]. There are both similarities and differences between these reports and our observations. In the case of nitrogen mustard-like and cisplatin ICLs, it was found that when forks converge at the ICL, their leading strands first stall 20–40 nt from the lesion before advancing to 1 nt from the crosslinked base [18]. This initial pausing was suggested to reflect the inherent size of the replisome's footprint on the DNA. In that study, plasmid DNAwas replicated by sequential incubation into cytosolic and nucleoplasmic extracts, a system that bypasses the need for nuclear assembly and results in a more synchronous and more efficient plasmid replication than our more physiological, nuclear assembly-dependent replication system. In the time course study shown on Figure 2 of ref. [18], the bands corresponding to leading strands after the initial pausing and after advancing to the ICL appear of similar intensity, even though they accumulate at different time points. Since we are looking at an asynchronous population of replicating molecules, we should have been able to detect both species if they existed in our system. Since we did not detect any pause until the leading strand polymerase reached the lesion, our results suggest that the psoralen ICL is more permissive to replisome progression. Cisplatin induces a significant deformation of the DNA helix by unwinding and bending the DNA [39], [40]. The nitrogen mustard-like agent was designed to minimize the distortion of the DNA double helix and to be located in the major groove of DNA [41]. In contrast, psoralen intercalates into the DNA and unwinds it but it neither bends DNA nor obstructs its major groove [42], [43]. These specific features may allow the progression of the replicative helicase despite the impossibility to unwind the two parental strands at the psoralen ICL.

Whether ICL repair in Xenopus egg extracts is exclusively coupled with DNA replication has been recently questioned [19]. Using the cytosolic + nucleoplasmic extract system, Raschle et al. [18] found that repair of a nitrogen-mustard like ICL was abolished by treatment with p27^Kip^, an inhibitor of origin firing, whereas Ben-Yehoda et al. [19] found that repair of an MMC-like ICL was resistant to geminin and roscovitin, which respectively inhibit origin licensing and activation. In our egg extract system, replication only occurs after a lag period during which plasmid DNA is chromatinized and assembled into pseudonuclei. If a large fraction of these plasmid DNA molecules underwent repair during this lag period, we would have observed a larger fraction of normal replication intermediates than the one observed in Figure 2B. For example, a complete bubble arc was never observed, which shows that most plasmid molecules still harbored the site-specific psoralen ICL when they underwent replication. This was true whether RIs were analyzed at 50, 90 or 120 min (data not shown). Although these results do not exclude the possibility of replication-independent ICL repair, most of the repair events we observed were coupled with DNA replication. The discrepancy with the results of Ben-Yehoda et al. [19] could be explained by a lack of prolonged exposure to concentrated nuclear proteins in our experiments, or by the use of different ICL substrates. Since Raschle et al. [18] also used concentrated nucleoplasmic extracts but did not observe replication-independent repair, we favor the latter interpretation.

## Materials and Methods

### Introducing a single psoralen ICL in a plasmid using a cleavable TFO

A modified TFO containing 5-methyldeoxycytidines instead of deoxycytidines and 5-propynyl-deoxyuridines instead of thymidines to increase triplex formation at neutral pH and linked in 5′ through a scissile S-S bond to 4,5′,8-trimethylpsoralen and in 3′ to biotin TEG was synthesized (PSO-SS-BIOT, purchased from Eurogentec, see structure in Fig. 1 A). 3.5 pmol (7 µg) of plasmid pTUC [44], containing a unique triplex helix site, were incubated overnight at 4°C with 300 pmol of PSO-SS-BIOT in 30 µL of 50 mM Tris-Cl, pH 6.8, 10 mM MgCl_2_, 60 mM KCl. Crosslinking was induced by a 10 min irradiation at 50 mW/cm^2^ under tungsten lamp through a pyrex filter to remove radiation below 310 nm. Noncrosslinked PSO-SS-BIOT was removed by heating at 65°C for 10 min and filtration through microcon YM-30 (Millipore).

### Purification of crosslinked plasmid

Plasmid purification was performed with ÄKTA 900 purifier system from GE Healtcare. pTUC-PSO-SS-BIOT was injected in a streptavidin column (Hitrap, GE Healtcare) pre-equilibrated with 20 mM Tris-Cl, pH 8.5, 50 mM NaCl, incubated overnight with the same buffer plus 50 mM DTT and eluted in buffer without DTT. The recovered pTUC-PSO was subjected to purity analysis by denaturing gel electrophoresis and q-PCR (see below).

### Denaturing gel electrophoretic analysis of crosslinked products

Crosslinked samples were digested with BamHI + EcoRI (New England Biolabs), filled with [α-^32^P]-dATP using AMV reverse transcriptase as described by the manufacturer (Finnzyme). Samples were heated for 3 min at 95°C and loaded onto a pre-warmed denaturing 10% polyacrylamid gel (19∶1 acrylamide/bis acrylamide) containing 7 M Urea in 1X TBE.

### Q-PCR analysis of crosslinked products

Using Brilliant SYBR Green Q-PCR kit (Strategene) with MX 3000 P system (Stratagene), PCR quantitation of plasmid crosslinking was performed using primers bought from Eurogentec. Primers 5′- TCG AGG TCG ACG GTA TCG ATA AG -3′ and 5′- GGC CGC TCT AGA ACT AGT GGA TC -3′ (P primers) flanked the ICL. Primers 5′- CAC GAG TGG GTT ACA TCG AAC TGG -3′ and 5′- CAA TAC GGG ATA ATA CCG CGC CAC -3′ (A primers) flanked a control region on the plasmid (see Fig. 1B). Samples were heated for 10 min at 95°C, followed by 37 cycles of 30 s at 95°C, 1 min at 56°C, 1 min at 72°C, the run was ended with one cycle of 1 min at 95°C, 30 s at 56°C and 30 s at 95°C. The percentage of Q-PCR inhibition at the crosslink site was 1-*P/A* where *P* and *A* represent the amount of amplified DNA at the crosslink site and at the control region, respectively.

### Plasmid replication in Xenopus egg extracts

Interphase egg extracts were prepared as previously described [45]. 250 ng of plasmid were incubated in 50 µL of Xenopus egg extracts supplemented with an energy regeneration mix (7.5 mM creatine phosphate, 1 mM ATP, 0.1 mM EGTA pH 7.7, 1 mM MgCl_2_), cycloheximid (250 µg/mL) and cytochalasin B (10 µg/mL) in the presence of radioactive [α-^32^P]-dATP. The reaction was stopped with 1 vol STOP buffer (EDTA 80 mM, NaCl 600 mM, SDS 2%), digested with RNAse A followed by proteinase K at 37°C. DNA was extracted using DNAzol (Invitrogen) and digested with AflIII (Biolabs). Caffeine (Sigma-Aldrich) was freshly dissolved to 100 mM in 10 mM Pipes pH 7.4 before each experiment. UCN-01 (Sigma-Aldrich) was dissolved to 1 mM in DMSO and stored at -20°C. Final drug concentrations are indicated in the Figure legends. KU55933 (ATM inhibitor) and C3742 (Chk2 inhibitor) were dissolved to 10 mM in DMSO and used at 10 µM in Xenopus egg extracts.

### Two-dimensional agarose gel electrophoresis

2D-gels were performed essentially as described [30]. Briefly, the first electrophoresis was in a 0.4% (w/v) agarose gel in 1X TBE buffer (89 mM Tris–borate, 2 mM EDTA) at 0.8 V/cm for 18 h at room temperature. Gels were then stained with 0.3 µg/mL ethidium bromide. The second dimension was performed in presence of 0.3 µg/mL ethidium bromide, in 1.2% (w/v) agarose gel in 1X TBE at 4 V/cm for 14 h in a 4°C cold room with buffer recirculation. Gels were dried under vacuum and exposed to phosphorimager screens. Radioactive signal quantification was performed with ImageGauge.
